# Association between night-shift frequency and metabolic syndrome among healthcare workers: a moderated mediation analysis of occupational stress and sleep quality

**DOI:** 10.3389/fpubh.2026.1940688

**Published:** 2026-09-18

**Authors:** Yin Yang, Xida Wang, Rui Dong, Xiaodong Guan

**Affiliations:** Yuncheng Hospital Affiliated to Shanxi Medical University, Yuncheng, Shanxi, China

**Keywords:** healthcare workers, metabolic syndrome, moderated mediation, night-shift work, occupational stress, sleep quality

## Abstract

**Background:**

Night-shift work is highly prevalent among healthcare workers and has been associated with various adverse health outcomes, including metabolic syndrome (MetS). However, the psychosocial factors potentially involved in this association remain unclear. This study aimed to examine the association between night-shift frequency and MetS among healthcare workers and to explore the potential statistical indirect association through occupational stress, as well as whether the association between night-shift frequency and occupational stress varied according to sleep quality, providing scientific evidence for workplace health promotion and MetS prevention in healthcare settings.

**Methods:**

A hospital-based cross-sectional study involving 719 healthcare workers from a tertiary Grade A hospital in Shanxi Province, China, was conducted from January to March 2026. Participants were selected using stratified cluster sampling. Night-shift frequency, occupational stress, sleep quality, and relevant covariates were assessed using questionnaires, with occupational stress measured using the Effort-Reward Imbalance Questionnaire. MetS was determined based on physical examination and laboratory criteria. Multivariable logistic regression was used to evaluate the associations of night-shift frequency and occupational stress with MetS. Exploratory mediation analysis was performed to assess the statistical indirect association through occupational stress, and moderated mediation analysis was used to examine whether the association between night-shift frequency and occupational stress varied according to sleep quality.

**Results:**

Of the 719 healthcare workers, 124 (17.2%) had MetS. Compared with participants reporting 0 night shifts per month, those reporting 1–3 night shifts per month did not have significantly higher odds of MetS, whereas those reporting ≥4 night shifts per month had substantially higher adjusted odds (OR = 6.65, 95% CI 2.56–17.28). Exploratory mediation analysis showed a statistically significant indirect association through occupational stress in the ≥4 night shifts per month group (indirect effect 0.725, 95% CI 0.424–1.173), accounting for approximately 27.7% of the total association. The association between night-shift frequency and occupational stress was stronger at poorer sleep quality levels. In the ≥4-night-shift group, the conditional indirect effect increased from 0.350 (95% CI 0.149–0.647) at better sleep quality to 1.187 (95% CI 0.699–1.921) at poorer sleep quality.

**Conclusion:**

Higher night-shift frequency was associated with higher odds of MetS among healthcare workers. Exploratory analyses suggested a potential statistical indirect association through occupational stress, and this indirect association varied according to sleep quality. These findings may help inform future workplace health strategies focusing on night-shift scheduling, occupational stress management, metabolic health monitoring, and sleep health support.

## 1 Introduction

Night-shift work is common among healthcare workers because continuous patient care requires hospitals to operate around the clock. In China, a substantial proportion of healthcare workers are regularly exposed to night shifts, often with high frequency and limited recovery time (1, 2). Frequent night-shift work has been associated with a range of adverse health outcomes, including metabolic abnormalities and metabolic syndrome (MetS) (3). MetS, characterized by central obesity, elevated blood pressure, hyperglycemia, and dyslipidemia, is associated with increased risks of cardiovascular disease and type 2 diabetes (4). Among healthcare workers, preventing MetS is important not only for long-term health but also for maintaining work capacity, workforce stability, and continuity of healthcare services.

Previous studies have suggested that night-shift work is associated with MetS and its individual components, with greater frequency and longer duration potentially linked to higher metabolic risk (3). Existing studies have primarily focused on biological pathways, including circadian rhythm disruption (5), altered melatonin secretion (6), and impaired insulin sensitivity. Nevertheless, night shifts are not merely a change in working hours; they are often accompanied by increased workload, emergency tasks, decision-making pressure, insufficient rest and recovery, and intensified work-family conflict. Thus, the role of modifiable psychosocial workplace factors in the association between night-shift work and metabolic health warrants further investigation.

Occupational stress may be one psychosocial factor potentially involved in this association. According to the effort-reward imbalance model, persistent stress may arise when high levels of work effort are not matched by adequate financial, social, or career-related rewards (7). This is particularly relevant to healthcare workers, who often face high work intensity, responsibility, and emotional demands. Frequent night shifts may contribute to greater perceived effort-reward imbalance by disrupting recovery, social support, and work-family balance (8). Occupational stress is an identifiable, assessable, and potentially modifiable psychosocial workplace factor (9). Prolonged occupational stress may contribute to metabolic abnormalities through neuroendocrine and inflammatory pathways, including activation of the hypothalamic-pituitary-adrenal axis, increased cortisol secretion, insulin resistance, and chronic low-grade inflammation (10, 11). Previous research has reported significant associations between occupational stress and individual components of MetS (12). Thus, occupational stress may be involved in the association between night-shift frequency and MetS.

The association between night-shift work and occupational stress may also vary according to sleep quality. Individuals with poorer sleep quality may have reduced recovery capacity and may be more susceptible to fatigue, emotional dysregulation, and heightened stress following frequent night shifts (13). In contrast, better sleep quality may be associated with better recovery and lower stress responses under irregular work schedules. This suggests that the association between night-shift frequency and occupational stress may vary according to sleep quality, and that the conditional indirect association with MetS may also differ across sleep quality levels. As sleep quality can be assessed in routine occupational assessments, it may help identify workers who are more vulnerable to night-shift-related metabolic risk.

In summary, this study examined the association between night-shift frequency and MetS among healthcare workers in a tertiary Grade-A hospital in China. It further explored the potential statistical indirect association through occupational stress and whether the association between night-shift frequency and occupational stress varied according to sleep quality. By focusing on potentially modifiable workplace-related and sleep-related factors, the findings may help identify healthcare workers vulnerable to night-shift-related metabolic risk and inform future research on workplace health strategies related to shift scheduling, psychosocial working conditions, and sleep health support. The proposed conceptual model is presented in Figure 1.

**Figure 1:**
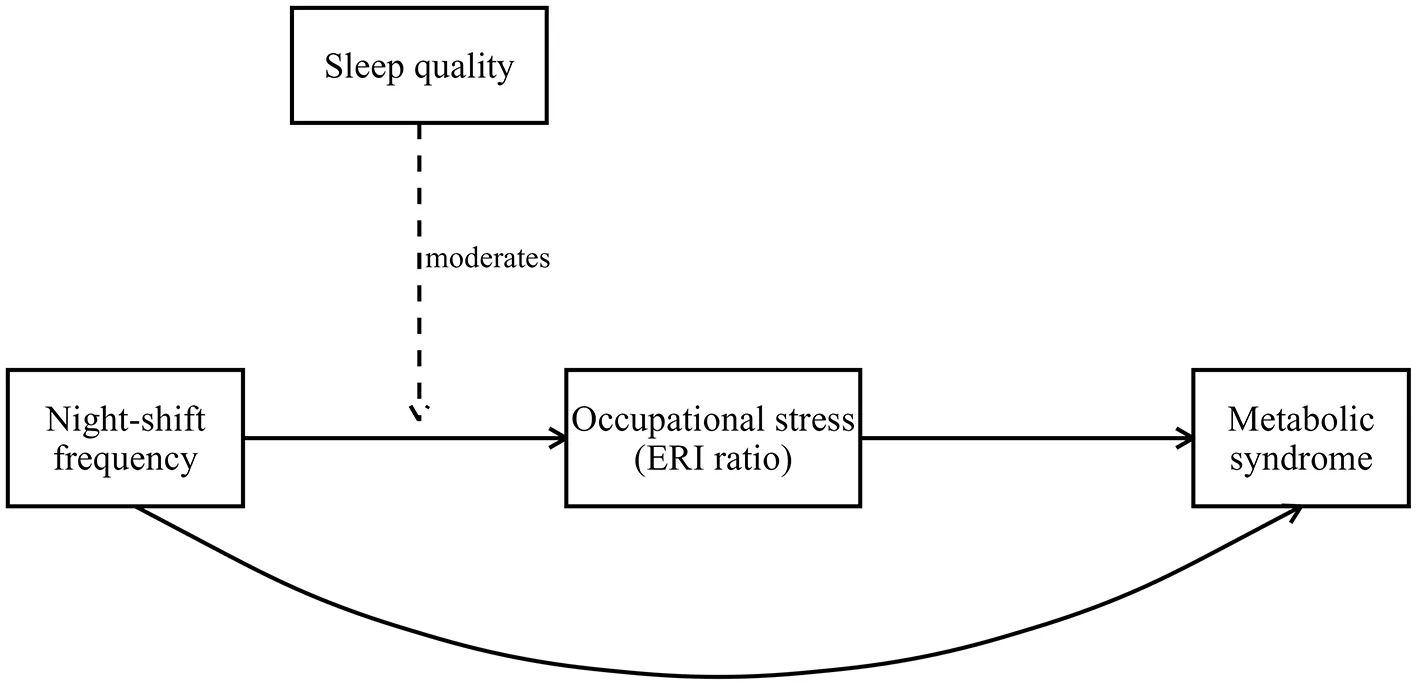
Conceptual model of the moderated mediation analysis.

## 2 Materials and methods

### 2.1 Study participants

This cross-sectional study was conducted from January to March 2026 in a tertiary Grade-A hospital in Shanxi Province, China. Stratified cluster sampling was used. Healthcare workers were stratified into physicians, nurses, medical technicians, and administrative or logistics staff. Departments were randomly selected within each stratum, and all eligible staff in selected departments were invited to participate. Inclusion criteria: (1) ≥1 year of continuous employment at the hospital; (2) voluntary participation with signed informed consent. Exclusion criteria: (1) severe chronic diseases, including malignant tumors, severe cardiovascular or cerebrovascular diseases, or hepatic or renal failure; (2) pregnancy or lactation; (3) regular use of medications affecting glucose or lipid metabolism within the past 3 months; and (4) missing data on core study variables. Biochemical indicators were extracted from clinical test reports generated by the hospital' s Health Examination Center during the survey period and anonymously linked to questionnaire data by authorized researchers.

The required sample size for this cross-sectional study was primarily estimated based on the precision of the overall detection rate of MetS among healthcare workers, using a commonly applied sample size calculation method for prevalence studies (14). The sample size formula for a single-population proportion was used:$n=\frac{{Z_{\alpha /2}}^{2}P\left(1-P\right)}{\delta^{2}}$. Based on previous literature (15) and a pilot survey, the prevalence of MetS among healthcare workers was approximately 14%. With α = 0.05, a margin of error of 0.05 and an expected response rate of 80%, at least 232 participants were needed. To further evaluate the adequacy of the sample size for the primary logistic regression analysis, an additional sample size estimation was performed using the method proposed by Hsieh et al. (16). The comparison between participants with ≥4 night shifts per month and those with 0 night shifts per month was used as the primary effect contrast. The expected effect size was based on a previous systematic review and meta-analysis of healthcare workers, which reported an OR of 2.17 (3). Based on a previous hospital-based study, the probability of MetS in the reference group was set at 10.3%, and the proportion of participants with high-frequency night-shift exposure was set at 58.6% (17). With a two-sided α = 0.05 and 80% power, at least 452 valid participants were estimated to be required. We invited 751 healthcare workers and obtained 719 valid questionnaires (response rate: 95.7%), satisfying the sample size requirement.

### 2.2 Data collection

A self-designed questionnaire was used to collect baseline information, including: (1) demographic characteristics, such as sex, age, ethnicity, educational level, marital status, and annual income; (2) lifestyle factors, such as smoking, alcohol consumption, physical activity, and dietary habits; (3) occupational factors, such as work experience, professional title, occupational category, and night-shift frequency; and (4) disease-related information, such as family history of metabolic diseases, including hypertension, hyperlipidemia, and coronary heart disease.

### 2.3 Definitions of relevant indicators

MetS was defined according to the International Diabetes Federation (IDF) (18). Participants were classified as having MetS if they had central obesity, defined as a waist circumference ≥90 cm in men or ≥80 cm in women, plus any two of the following four components: (1) triglycerides (TG) ≥1.7 mmol/L or currently receiving treatment for this condition; (2) high-density lipoprotein cholesterol (HDL-C) < 1.03 mmol/L in men or < 1.29 mmol/L in women or currently receiving treatment for this condition; (3) blood pressure ≥130/85 mmHg or treatment for previously diagnosed hypertension and currently receiving treatment; (4) fasting plasma glucose ≥5.6 mmol/L or a confirmed diagnosis of type 2 diabetes.

Night-shift work was defined with reference to the International Agency for Research on Cancer (IARC)(19), adapted to the scheduling practices of Chinese healthcare institutions as night duty, night-shift, or rotating-shift work occurring outside standard daytime hours (08:00–18:00) and overlapping any part of the period between 23:00 and 07:00. The average number of night shifts per month during the previous 3 months was used to assess recent night-shift exposure frequency.

Previous occupational health studies among healthcare workers have used the number of night shifts per month to assess night-shift exposure (20). However, there are currently no standardized cutoffs for classifying night-shift frequency, and different studies have adopted varying classification methods based on their research objectives, scheduling systems, and sample distributions. During the survey design stage, based on previous studies among nurses and healthcare workers (21, 22) and the scheduling characteristics of the study hospital, night-shift frequency was initially classified into four categories: 0, 1–3, 4–7, and ≥8 night shifts per month. Specifically, 0 night shifts per month indicated no recent exposure to night shifts; 1–3 night shifts per month represented relatively low-frequency exposure, corresponding approximately to fewer than one night shift per week; and ≥4 night shifts per month corresponded approximately to at least one night shift per week and represented relatively high-frequency exposure.

In the actual survey, 59 participants (8.21% of the total sample) reported ≥8 night shifts per month. Given the relatively small proportion of participants in this group, retaining the four-category classification would have resulted in an uneven distribution of sample sizes across the exposure groups and could have reduced the precision of effect estimates for the highest-frequency group. Considering the study aim and previous studies (17) that used 4 or ≥4 night shifts per month as a basis for defining night-shift exposure, the 4–7 and ≥8 categories were combined. Night-shift frequency was therefore ultimately classified as 0, 1–3, and ≥4 night shifts per month, representing no recent night-shift exposure, relatively low-frequency night-shift exposure, and relatively high-frequency night-shift exposure, respectively. This categorization was intended to reflect practical scheduling patterns while providing relatively stable statistical estimates and does not imply that 4 night shifts per month is an established biological threshold or a definitive cutoff for a dose-response relationship.

Occupational stress was measured using the Effort-Reward Imbalance (ERI) Questionnaire (23), which consists of 23 items covering three dimensions: effort, reward, and overcommitment. The ERI model was originally proposed by Siegrist. The Chinese version of the ERI-Q has been validated among Chinese healthcare workers, with Cronbach's α coefficients of 0.78, 0.81, and 0.74 for the effort, reward, and overcommitment dimensions, respectively, demonstrating good internal consistency and psychometric properties (24). A 5-point Likert scale was used, with “1” indicating “strongly disagree” and “5” indicating “strongly agree”; the item scores within each dimension were summed to obtain the corresponding dimension score. The effort-reward ratio was calculated as follows: effort dimension score / (reward dimension score × 6/11). An ERI ratio ≤ 1 indicated effort–reward balance, whereas an ERI ratio >1 indicated effort–reward imbalance. In the present study, Cronbach's α coefficients for the effort, reward, and over commitment dimensions were 0.853, 0.887, and 0.814, respectively, and the Kaiser-Meyer-Olkin (KMO) value was 0.917.

A research-specific sleep quality score was developed using selected items related to the Pittsburgh Sleep Quality Index (PSQI) (25). In this study, sleep quality was primarily used as a moderator to explore individual differences in the association between night-shift frequency and occupational stress, rather than for the clinical screening or diagnosis of sleep disorders. To reduce the response and recall burden among healthcare workers and enhance the feasibility of the field survey (26, 27), the full PSQI was not administered in this study. Compared with the standard PSQI, this study did not calculate habitual sleep efficiency separately and simplified items related to sleep disturbances and daytime dysfunction. In the standard PSQI, habitual sleep efficiency is calculated based on actual sleep duration and time in bed, while time in bed requires information such as usual bedtime and wake time. Given that the sleep timing of shift-working healthcare workers may vary according to their work schedules, further collecting and summarizing their “usual” bedtime, wake time, and actual sleep duration would increase the burden of time-related data collection and recall. To keep the questionnaire concise, the sleep disturbance section primarily retained “nighttime awakenings” to reflect sleep maintenance, while the daytime dysfunction section selected “daytime sleepiness” to reflect the degree to which daytime alertness is impaired. Based on the PSQI assessment framework and previous studies on shift work and sleep (28, 29), this study selected six indicators: sleep duration, sleep latency, nighttime awakenings, self-rated sleep quality, daytime sleepiness, and use of sleep medication. These indicators reflect, respectively, sleep quantity, sleep initiation, sleep maintenance, subjective sleep experience, daytime alertness, and coping behaviors related to sleep problems. Each item was scored on a scale of 0–3, with a total score ranging from 0 to 18; higher scores indicated poorer sleep quality. Cronbach's α for this score was 0.771, indicating acceptable internal consistency. Exploratory factor analysis revealed a KMO value of 0.780, with Bartlett's test of sphericity yielding χ^2^ = 1107.915, df = 15, *P* < 0.001. Principal axis factoring extracted a single factor, with factor loadings for individual items ranging from 0.330 to 0.749, providing some preliminary support for a common underlying structure. In this study, the score was used as a continuous measure to reflect the relative sleep status within the sample; standard PSQI cutoff values were not used to classify sleep quality.

### 2.4 Physical examination and laboratory tests

With authorization from the hospital, physical examination reports from the same period were retrieved from the hospital information system. Physical examination indicators included height, weight, waist circumference, and blood pressure, which were measured by trained medical professionals using standardized procedures. Laboratory test indicators included fasting plasma glucose, triglycerides, total cholesterol, low-density lipoprotein cholesterol, and high-density lipoprotein cholesterol. Participants fasted for at least 8 h before blood sampling. MetS and its components were determined according to the IDF diagnostic criteria based on objective physical examination and laboratory test results.

### 2.6 Statistical methods

All statistical analyses were performed using R version 4.5.2. Continuous variables that followed a normal distribution are presented as mean ± SD, and comparisons among the three night-shift frequency groups were performed using one-way analysis of variance (ANOVA); Welch's ANOVA was used when the assumption of homogeneity of variance was not met. Continuous variables with a skewed distribution are presented as median and interquartile range [M (P25, P75)], and group comparisons were performed using the Kruskal–Wallis H test. Categorical variables are presented as frequencies and proportions (%), and group comparisons were performed using Pearson's chi-square test or Fisher's exact test, as appropriate.

Multivariable logistic regression was used to assess the associations of night-shift frequency and occupational stress with MetS, with results reported as odds ratios (ORs) and 95% confidence intervals (CIs). Night-shift frequency was categorized as 0, 1–3, or ≥4 night shifts per month; with 0 night shifts per month used as the reference group. Potential confounders were selected based on previous literature, professional knowledge, and their potential associations with both exposure and outcome (15, 30), including sex, age, ethnicity, physical activity, occupational category, healthy diet score, professional title, marital status, smoking, alcohol consumption, annual income, and family history of metabolic disease. Sensitivity analysis modeled night-shift frequency as an ordinal variable to test for linear trends. Because MetS was not a rare outcome, Poisson regression with robust variance was further performed as a sensitivity analysis to estimate prevalence ratios (PRs) and 95% CIs.

Exploratory mediation analysis was conducted to assess the potential statistical indirect association between night-shift frequency and MetS through occupational stress. Night-shift frequency was specified as the exposure (X), occupational stress as the mediator (M), and MetS as the outcome (Y). The mediator was modeled using linear regression and the outcome using logistic regression, with adjustment for the covariates included in the primary analysis. Total, direct, and indirect effects were estimated for each night-shift frequency group relative to the 0 night shifts per month.

To examine moderated mediation, an interaction model was used to assess whether the association between night-shift frequency and occupational stress varied according to sleep quality. Sleep quality was treated as a continuous variable and mean-centered before analysis. The interaction between night-shift frequency and sleep quality was tested, and conditional indirect associations between night-shift frequency and MetS through occupational stress were estimated at different levels of sleep quality, namely the mean and ± 1 SD. The bootstrap method with 5,000 resamples was used to calculate 95% CIs for the conditional indirect associations. A conditional indirect association was considered statistically significant if the 95% CI did not include 0. All statistical tests were two-sided, and P < 0.05 was considered statistically significant.

### 2.7 Quality control and assessment of common method bias

Questionnaires were administered through Wenjuanxing, with instructions and study purpose provided on the first page. After collection, self-reported data and objective examination data were independently checked and organized by two trained researchers.

Because exposure, psychosocial, and behavioral variables were self-reported, Harman's single-factor test was conducted. Six factors had eigenvalues >1, and the first explained 26.31% of variance (< 40%), suggesting that substantial common method bias was unlikely.

## 3 Results

### 3.1 Baseline characteristics of participants

Among the 719 participants, 140 (19.47%), 180 (25.03%), and 399 (55.50%) reported 0, 1–3, and ≥4 night shifts per month, respectively. Significant differences were observed among the three groups in age, marital status, educational level, annual income, work experience, occupational category, professional title, effort-reward ratio, effort-reward imbalance, sleep quality score, healthy diet score, smoking status, waist circumference, and HDL-C (all *P* < 0.05). No significant differences were observed in the remaining baseline characteristics (all *P* > 0.05). Details are shown in Table 1.

**Table 1:** Comparison of baseline characteristics of healthcare workers across different night-shift frequency groups (*n* = 719).

Variable	Total sample (*n* = 719)	0 night shifts/month (*n* = 140)	1–3 night shifts/month (*n* = 180)	≥4 night shifts/month (*n* = 399)	Statistic (F/H/χ^2^)	*P*
Age (years)	36.97 ± 8.52	42.01 ± 10.22	36.86 ± 7.77	35.25 ± 7.44	26.089	< 0.001
Sex					5.093	0.078
Male	225 (31.29)	33 (23.57)	57 (31.67)	135 (33.83)		
Female	494 (68.71)	107 (76.43)	123 (68.33)	264 (66.17)		
Ethnicity					–	0.404
Han Chinese	713 (99.17)	139 (99.29)	180 (100.00)	394 (98.75)		
Others	6 (0.83)	1 (0.71)	0 (0.00)	5 (1.25)		
Marital status					–	0.005
Single	127 (17.66)	14 (10.00)	29 (16.11)	84 (21.05)		
Married/ living with a partner	580 (80.67)	122 (87.14)	146 (81.11)	312 (78.20)		
Divorced/ widowed	12 (1.67)	4 (2.86)	5 (2.78)	3 (0.75)		
Education level					–	< 0.001
Associate degree or below	19 (2.65)	9 (6.43)	4 (2.22)	6 (1.50)		
Bachelor's degree	497 (69.12)	104 (74.29)	140 (77.78)	253 (63.41)		
Master's degree	194 (26.98)	26 (18.57)	33 (18.33)	135 (33.83)		
Doctorate	9 (1.25)	1 (0.71)	3 (1.67)	5 (1.25)		
Annual income (RMB)					–	0.026
< 100,000	304 (42.28)	64 (45.71)	68 (37.78)	172 (43.11)		
100,000–200,000	398 (55.36)	68 (48.57)	108 (60.00)	222 (55.64)		
200,000–300,000	17 (2.36)	8 (5.71)	4 (2.22)	5 (1.25)		
Work experience (years)	12.87 ± 9.57	19.34 ± 11.83	13.09 ± 8.37	10.50 ± 8.02	35.302	< 0.001
Occupational category					110.998	< 0.001
Physician	242 (33.66)	25 (17.86)	35 (19.44)	182 (45.61)		
Nurse	355 (49.37)	63 (45.00)	102 (56.67)	190 (47.62)		
Medical technician	46 (6.40)	15 (10.71)	21 (11.67)	10 (2.51)		
Administrative staff	76 (10.57)	37 (26.43)	22 (12.22)	17 (4.26)		
Professional title					29.947	0.038
None	31 (4.31)	6 (4.29)	6 (3.33)	19 (4.76)		
Junior	170 (23.64)	26 (18.57)	39 (21.67)	105 (26.32)		
Intermediate	336 (46.73)	51 (36.43)	90 (50.00)	195 (48.87)		
Associate senior	147 (20.45)	42 (30.00)	40 (22.22)	65 (16.29)		
Senior	35 (4.87)	15 (10.71)	5 (2.78)	15 (3.76)		
Effort-reward ratio	0.98 ± 0.29	0.84 ± 0.24	0.85 ± 0.27	1.09 ± 0.27	79.434	< 0.001
Effort-reward imbalance					83.444	< 0.001
Imbalance	337 (46.87)	33 (23.57)	57 (31.67)	247 (61.90)		
Balance	382 (53.13)	107 (76.43)	123 (68.33)	152 (38.10)		
Sleep quality score	7.25 ± 4.06	5.24 ± 3.35	6.76 ± 3.55	8.18 ± 4.20	35.452	< 0.001
Healthy diet score	3.95 ± 1.51	4.26 ± 1.35	3.91 ± 1.59	3.86 ± 1.52	3.853	0.022
Physical activity level					3.964	0.411
Low (< 600)	494 (68.71)	90 (64.29)	129 (71.67)	275 (68.92)		
Moderate (600–2,999)	203 (28.23)	43 (30.71)	48 (26.67)	112 (28.07)		
High (≥3,000)	22 (3.06)	7 (5.00)	3 (1.67)	12 (3.01)		
Smoking status					13.362	0.010
Never smoker	636 (88.46)	131 (93.57)	153 (85.00)	352 (88.22)		
Former smoker	33 (4.59)	3 (2.14)	16 (8.89)	14 (3.51)		
Current smoker	50 (6.95)	6 (4.29)	11 (6.11)	33 (8.27)		
Alcohol consumption					4.501	0.342
Never drinker	561 (78.03)	116 (82.86)	143 (79.44)	302 (75.69)		
Former drinker	21 (2.92)	4 (2.86)	3 (1.67)	14 (3.51)		
Current drinker	137 (19.05)	20 (14.29)	34 (18.89)	83 (20.80)		
Family history					5.648	0.059
Yes	437 (60.78)	95 (67.86)	114 (63.33)	228 (57.14)		
No	282 (39.22)	45 (32.14)	66 (36.67)	171 (42.86)		
BMI (kg/m^2^)	23.57 ± 3.63	23.46 ± 3.20	23.14 ± 3.28	23.81 ± 3.90	2.344	0.097
Waist circumference (cm)^a^	76.00 (70.00, 87.00)	75.50 (70.00, 85.25)	75.00 (70.00, 83.00)	78.00 (70.00, 89.00)	6.623	0.036
SBP (mmHg)	113.93 ± 12.20	113.74 ± 13.23	112.16 ± 11.60	114.80 ± 12.02	2.940	0.053
DBP (mmHg)	74.65 ± 8.75	74.08 ± 8.25	73.57 ± 8.98	75.34 ± 8.77	2.907	0.055
FPG (mmol/L)^a^	4.60 (4.26, 5.00)	4.60 (4.29, 4.93)	4.61 (4.27, 4.98)	4.60 (4.24, 5.04)	0.144	0.931
HDL-C (mmol/L)	1.33 ± 0.32	1.40 ± 0.36	1.38 ± 0.28	1.29 ± 0.31	8.949	< 0.001
TG (mmol/L)^a^	0.97 (0.69, 1.44)	1.04 (0.69, 1.53)	0.90 (0.69, 1.24)	0.97 (0.70, 1.58)	4.456	0.108

^a^Variables with a non-normal distribution are presented as median (P25, P75).

### 3.2 Detection rates of MetS and its components by night-shift frequency

The detection rate of metabolic syndrome in this study was 17.2%. MetS detection rates differed significantly across groups (χ^2^ = 58.125, *P* < 0.001), with rates of 7.1%, 3.9%, and 26.8% in the 0, 1–3, and ≥4 night shifts per month groups, respectively. The trend test was statistically significant (P for trend < 0.001). The detection rates of abdominal obesity, elevated blood pressure, elevated fasting plasma glucose, elevated triglycerides, and low HDL-C also differed significantly among groups (all *P* < 0.05). Details are shown in Table 2.

**Table 2:** Detection rates of MetS and its components among healthcare workers by night-shift frequency.

Indicators	Frequency of night shifts			Statistic		
	**0 night shifts/month**	**1–3 night shifts/month**	≥**4 night shifts/month**	χ^2^	***P-*** **value**	**Trend** ***P-*****value**
MetS	10 (7.1)	7 (3.9)	107 (26.8)	58.125	< 0.001	< 0.001
Abdominal obesity	40 (28.6)	38 (21.1)	144 (36.1)	13.473	0.001	0.014
Elevated blood pressure	25 (17.9)	28 (15.6)	111 (27.8)	13.018	0.001	0.002
Elevated fasting plasma glucose	10 (7.1)	14 (7.8)	60 (15.0)	9.809	0.007	0.004
Elevated triglycerides	23 (16.4)	19 (10.6)	90 (22.6)	12.351	0.002	0.017
Low HDL-C	41 (29.3)	43 (23.9)	174 (43.6)	24.255	< 0.001	< 0.001

Count data are expressed as *n* (%). Comparisons between groups were performed using the chi-square test, and trend analysis was conducted using the linear trend chi-square test.

### 3.3 Associations of night-shift frequency and occupational stress with MetS

After adjustment for age, sex and other covariates in a multivariable logistic regression model 3, the 1–3 night shifts per month group showed no statistically significant difference, whereas the ≥4 night shifts per month group had an *OR* of 12.969 (*95*% *CI*: 5.298–31.743). Further adjustment for ERI ratio attenuated but did not eliminate this association (OR = 6.651, 95% *CI*: 2.560–17.279). Treating night-shift frequency as ordinal revealed a significant dose-response relationship (*P* for trend < 0.001). Additionally, each 0.1-unit increase in ERI ratio was associated with a 31.3% higher odds of MetS (*OR* = 1.313, 95% *CI*: 1.186–1.452). Detailed results are presented in Table 3 and Supplementary Table 1.

**Table 3:** Multivariable logistic regression analysis of the associations of night-shift frequency and occupational stress with MetS [OR (95% CI)].

**Variables**	Model 1	Model 2	Model 3	Model 4
Sex	–	0.3650 (0.234–0.568)^***^	0.425 (0.220–0.822)^**^	0.387 (0.196–0.763)^*^
Age (years)	–	1.099 (1.067–1.133)^***^	1.163 (1.096–1.233)^***^	1.149 (1.081–1.221)^***^
Night shift frequency				
0 night shift/month	1.000	1.000	1.000	1.000
1–3 night shifts/month	0.526 (0.195–1.419)	0.898 (0.315–2.558)	0.824 (0.273–2.485)	0.759 (0.240–2.404)
≥4 night shifts/month	4.764 (2.413–9.405)^***^	11.092 (4.975–24.730)^***^	12.969 (5.298–31.743)^***^	6.651 (2.560–17.279)^***^
Occupational stress (ERI ratio)	–	–		1.313 (1.186–1.452)^***^

Model 1 was unadjusted. Model 2 was adjusted for age and sex. Model 3 was further adjusted for educational level, ethnicity, physical activity, occupational category, healthy diet score, professional title, marital status, smoking, alcohol consumption, annual income, and family history of metabolic diseases. Model 4 was additionally adjusted for the ERI ratio. Occupational stress was reported as the *OR* corresponding to each 0.1-unit increase in the ERI ratio. ^*^P < 0.05, ^**^P < 0.01, ^***^P < 0.001.

### 3.4 Exploratory mediation analysis of occupational stress

After adjusting for sex, age, educational level and other covariates, exploratory bootstrap mediation analysis showed no statistically significant indirect association for participants reporting 1–3 night shifts per month. Among those reporting ≥4 night shifts per month, the estimated total association was 2.619 (95% CI: 2.023–3.890), the direct association was 1.895 (95% CI: 1.220–3.104), and the indirect association through occupational stress was 0.725 (95% CI: 0.424–1.173). The indirect association accounted for approximately 27.7% of the total association. These findings suggest a potential statistical indirect association between higher night-shift frequency and MetS through occupational stress. Details are shown in Table 4.

**Table 4:** Exploratory mediation analysis of occupational stress in the association between night-shift frequency and MetS.

Night-shift frequency	Path	Effect Size	standard error	95% CI	Effect size *(*%)
1–3 night shifts/month	Total effect	−0.206	0.869	−1.704–1.066	100.00
	Direct effect	−0.276	0.872	−1.773–0.974	133.61
	Indirect effect	0.069	0.093	−0.100–0.264	−33.61
≥4 night shifts/month	Total effect	2.619	0.531	2.023–3.890	100.00
	Direct effect	1.895	0.533	1.220–3.104	72.34
	Indirect effect	0.725	0.194	0.424–1.173	27.66

Effect estimates are model-derived regression coefficients. The 95% CIs were calculated using the bootstrap method with 5,000 resamples.

### 3.5 Conditional indirect effects of night-shift frequency on metabolic syndrome through occupational stress at different levels of sleep quality

Interaction analysis indicated that the association between night-shift frequency and occupational stress varied significantly according to sleep quality. Compared with workers reporting no night shifts, the interaction coefficients were 0.017 for those working 1–3 night shifts per month and 0.038 for those working ≥4 night shifts per month, indicating stronger associations between night-shift frequency and occupational stress at poorer sleep quality levels. The indices of moderated mediation were statistically significant for both the 1–3 night-shift group (index = 0.045, 95% CI: 0.007–0.103) and the ≥4 night-shift group (index = 0.103, 95% CI: 0.055–0.179). In the 1–3 night-shift group, the conditional indirect association through occupational stress was statistically significant only at poorer sleep quality (indirect effect = 0.338, 95% CI: 0.095–0.734). In the ≥4 night-shift group, the estimated conditional indirect association increased from 0.350 (95% CI: 0.149–0.647) at better sleep quality to 1.187 (95% CI: 0.699–1.921) at poorer sleep quality. These results are presented in Table 5, Supplementary Table 2, Figure 2, and Supplementary Figure 1.

**Table 5:** Conditional indirect effects of night-shift frequency on metabolic syndrome through occupational stress at different levels of sleep quality.

Night-shift frequency	Sleep quality level	Effect size	Standard error	95% CI	Interaction-mediation index (95% *CI*)
1–3 night shifts/month	Better (mean−1*SD*)	−0.029	0.114	−0.263–0.114	0.045 (0.007–0.103)
	Average (mean)	0.155	0.099	−0.004–0.381	
	Poorer (Mean+1*SD*)	0.338	0.163	0.095–0.734	
≥4 night shifts/month	Better (mean−1*SD*)	0.350	0.127	0.149–0.647	0.103 (0.055–0.179)
	Average (mean)	0.768	0.202	0.456–1.241	
	Poorer (Mean+1*SD*)	1.187	0.312	0.699–1.921	

**Figure 2:**
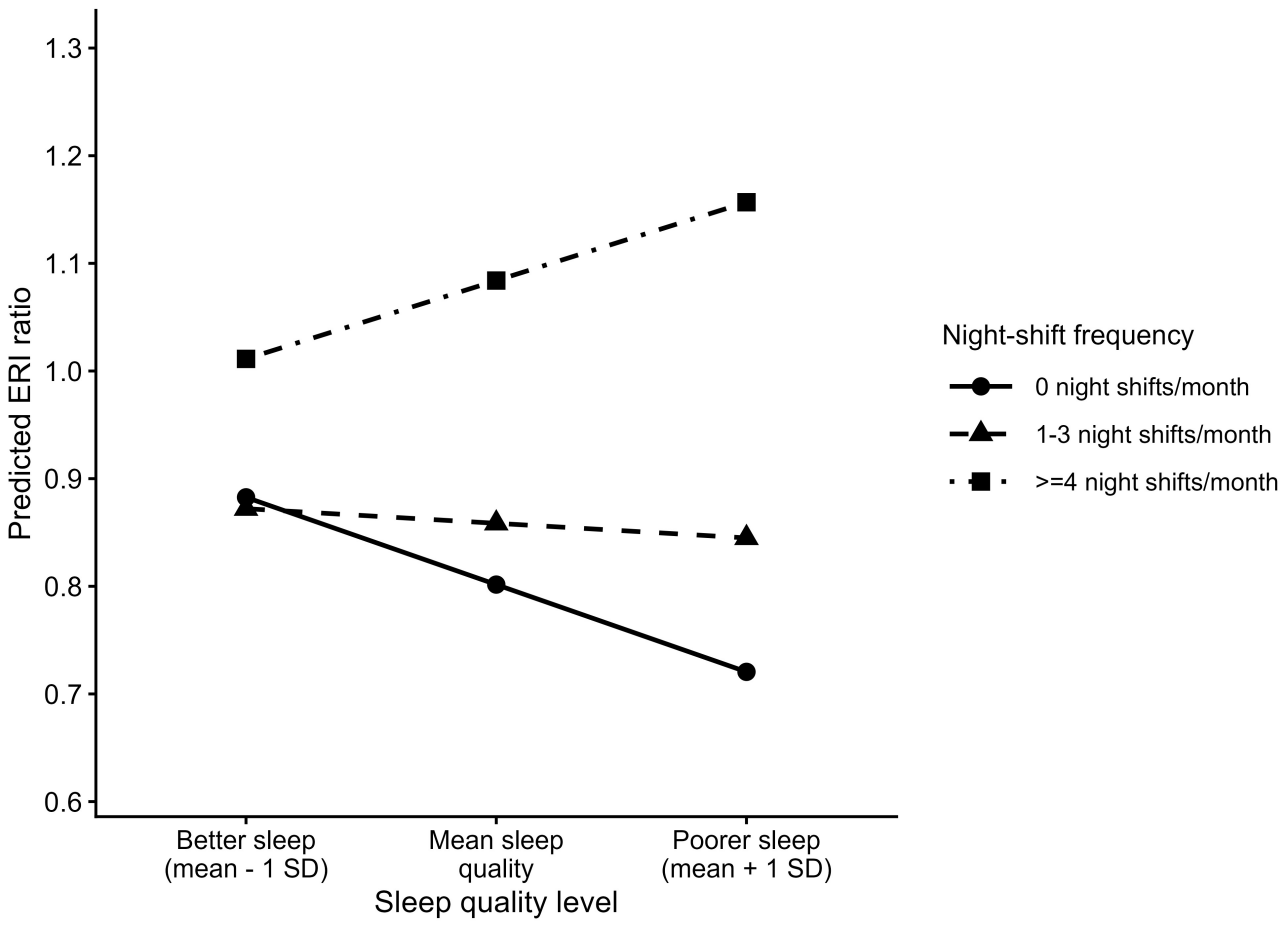
Association between night-shift frequency and occupational stress across different levels of sleep quality.

## 4 Discussion

In this hospital-based cross-sectional study, the detection rate of MetS among healthcare workers was 17.2%. Compared with workers reporting no night shifts, those reporting ≥4 night shifts per month had significantly higher odds of MetS, whereas no statistically significant association was observed among those reporting 1–3 night shifts per month. Exploratory mediation analysis showed a statistically significant indirect association between frequent night shifts and MetS through occupational stress, accounting for approximately 27.7% of the total association. Additionally, the association between night-shift frequency and occupational stress was stronger among participants with poorer sleep quality and was accompanied by a larger conditional indirect association with MetS. Together, these findings suggest that occupational stress and sleep quality may represent potentially modifiable factors relevant to night-shift-related metabolic health among healthcare workers. Given the cross-sectional design, these mediation and moderated mediation findings should be regarded as exploratory and hypothesis-generating and should not be interpreted as evidence of causal pathways.

The observed detection rate of MetS was higher than the 13.6% reported among healthcare workers in Nanjing in 2020 (15), but lower than the 24.5% reported in the general population (31). Differences across studies may be related to variations in sex composition, occupational category, regional characteristics, and diagnostic criteria. The association between frequent night shifts and MetS was broadly consistent with previous evidence linking night-shift work to metabolic abnormalities (30). Participants reporting ≥4 night shifts per month also had higher frequencies of elevated blood pressure, elevated fasting glucose, elevated triglycerides, and low high-density lipoprotein cholesterol, suggesting clustering of metabolic abnormalities. Potential mechanisms may involve circadian rhythm disruption. Nighttime artificial light may suppress pineal melatonin secretion (32), potentially impairing its antioxidant and lipid-regulatory roles (33). Concurrent sleep loss and inadequate recovery may dysregulate the HPA axis, alter cortisol rhythms, promote visceral fat accumulation, and contribute to insulin resistance and hypertension (34). Furthermore, altered ghrelin and leptin secretion may further disrupt glucose regulation (35), potentially contributing to MetS (36).

The association between ≥4 night shifts per month and MetS was relatively strong, but the magnitude of the OR should be interpreted cautiously. A previous systematic review and meta-analysis of healthcare workers reported a pooled OR of 2.17 (95% CI: 1.31–3.60) for the association between shift work and MetS (3), with substantial heterogeneity across studies (I^2^ = 82%), suggesting that differences in study populations, definitions of night-shift exposure, scheduling systems, and confounder adjustment may influence effect estimates. First, participants reporting no night shifts were older on average than those reporting ≥4 night shifts per month (42.01 vs. 35.25 years). Because age is closely associated with MetS, age and related factors may have acted as negative confounders, partially masking the positive association between night-shift frequency and MetS and contributing to a larger effect estimate after adjustment. Second, although the multivariable model adjusted for multiple demographic, occupational, and lifestyle factors, residual confounding cannot be excluded, as detailed information on the duration of individual night shifts, consecutive night shifts, shift patterns, overtime, and total working hours was unavailable (37, 38).

In addition, differences in the likelihood of entering or remaining in high-frequency night-shift work across age groups, occupations, and professional titles may have introduced selection effects. The “healthy shift worker effect” in occupational epidemiology may also suggest that individuals in poorer health or with lower tolerance for night work may reduce or discontinue night-shift work, thereby affecting the distribution of health characteristics across different night -shift groups (39). The overall detection rate of MetS in this study was 17.2%, and the detection rate was 26.8% in the ≥4 night shifts per month group, indicating that MetS was not a rare outcome in this study. Under these circumstances, ORs estimated from logistic regression may be numerically larger than the corresponding PRs. Previous methodological studies have indicated that, for common binary outcomes in cross-sectional studies, ORs may overestimate the corresponding PRs, whereas Poisson regression with robust variance can directly estimate adjusted PRs (40). Therefore, we performed a sensitivity analysis using Poisson regression with robust variance. In Model 4, logistic regression yielded an OR of 6.651 (95% CI: 2.560–17.279) for the ≥4 night shifts per month group, whereas Poisson regression with robust variance yielded a PR of 3.663 (95% CI: 2.117–6.338); the direction and statistical significance of the association remained consistent in both models. These results provide some support for the robustness of the association between frequent night-shift work and MetS. Nevertheless, the results should be interpreted as evidence of a strong statistical association rather than a causal increase in risk of the same magnitude, and the effect size requires further confirmation in prospective, multicenter studies.

Beyond the overall association, exploratory mediation analysis suggested a potential statistical indirect association between night-shift frequency and MetS through occupational stress. The proportion of participants with effort-reward imbalance was 46.9%, higher than the 26.5% reported by Kong et al. among clinical nurses (41), suggesting relatively high stress levels in this population. Frequent night shifts may disrupt work-family balance, weaken social support and increase perceived effort-reward imbalance, potentially contributing to sustained stress responses. Chronic occupational stress may activate the HPA axis and sympathetic adrenomedullary system (42), alter cortisol and catecholamine levels, and promote low-grade inflammation (10), which may contribute to disturbances in glucose and lipid metabolism. In the current statistical decomposition, the indirect association through occupational stress accounted for approximately 27.7% of the total association between night-shift frequency and MetS, suggesting that occupational stress may be involved in the observed association. Given the cross-sectional design, this finding should be interpreted as exploratory and hypothesis-generating and does not establish the temporal sequence or a causal mediation pathway among night-shift frequency, occupational stress, and MetS. Therefore, occupational stress may represent a potentially modifiable psychosocial factor worthy of further evaluation in prospective and intervention studies.

The association between night-shift frequency and occupational stress varied according to sleep quality, with a stronger association observed among participants with poorer sleep quality. A larger conditional indirect association with MetS through occupational stress was also observed at poorer sleep quality levels. Poorer sleep quality may reflect hyperarousal, heightened stress reactivity, and impaired recovery (43), while restorative sleep, particularly slow-wave sleep, may help attenuate excessive HPA axis responses after night-shift work (44). Therefore, sleep quality may help identify healthcare workers in whom the observed associations between night-shift frequency, occupational stress, and MetS are stronger.

These findings may have implications for MetS prevention and workplace health promotion among healthcare workers. Metabolic health strategies for night-shift workers may need to consider not only shift exposure but also occupational stress and sleep recovery. Potential organizational approaches for further evaluation include optimizing night-shift schedules according to workload and staffing levels, reducing consecutive night shifts, and ensuring adequate recovery time after night shifts. Fair resource allocation, timely performance feedback, and stronger managerial support may also help alleviate sustained effort-reward imbalance. Additionally, occupational stress and sleep quality assessments may be useful components of workplace health monitoring, while stress management, sleep hygiene education, psychological support, and lifestyle interventions could be explored as complementary approaches alongside organizational improvements in working conditions. Given the cross-sectional design of this study, these strategies should be regarded as potential directions for future intervention research rather than definitive recommendations. Their effectiveness in improving metabolic health should be confirmed in prospective or interventional studies before firm recommendations are made.

This study has several strengths. First, it incorporates night-shift frequency, occupational stress, and sleep quality into the same analytical framework to explore psychosocial factors potentially involved in the association between night-shift frequency and MetS. Second, it highlights occupational stress and sleep quality as identifiable, assessable, and potentially modifiable factors, which may provide useful insights for workplace health promotion in healthcare institutions. Third, stratified cluster sampling included participants from different occupational groups, and MetS was determined using objective physical examination measurements and laboratory test results, strengthening the reliability of outcome assessment.

This study has several limitations. First, the cross-sectional design precludes establishing the temporal sequence among night-shift frequency, occupational stress, sleep quality, and MetS and making causal inferences. Therefore, the findings from the mediation and moderated mediation analyses should be regarded as exploratory and hypothesis-generating rather than as evidence of definitive causal pathways. These findings require further validation in longitudinal studies. Second, participants were recruited from a single tertiary hospital, limiting generalizability. Third, night-shift exposure in this study was assessed primarily using participants' self-reported average number of night shifts per month during the previous 3 months, which may be subject to recall bias and exposure misclassification. Additionally, this study did not record the specific start and end times or duration of each night shift; therefore, total night-work hours could not be calculated. Future studies could incorporate objective hospital scheduling records and collect information on the duration of individual night shifts to enable a more accurate and comprehensive assessment of night-shift exposure. Fourth, this study did not use the full PSQI; instead, based on the study objectives and survey feasibility, a research-specific composite sleep quality score was constructed using selected PSQI-related items, which did not fully cover habitual sleep efficiency, sleep disturbances, and daytime dysfunction. Although this score showed acceptable internal consistency and some preliminary structural support, it has not undergone independent external or criterion validation; therefore, it cannot be considered equivalent to the standard PSQI, and this limitation also restricts direct comparisons with studies using the full PSQI. Future research should use complete, validated, standardized sleep assessment instruments to further verify these findings. Fifth, the relatively small numbers of MetS cases in some night-shift frequency groups may have contributed to wide 95% CIs for the OR estimates, indicating limited precision of the effect estimates.

In summary, frequent night-shift work among healthcare workers was associated with higher odds of MetS. Exploratory analyses suggested a potential statistical indirect association through occupational stress, and the association between night-shift frequency and occupational stress was stronger at poorer sleep quality levels. These findings may inform future workplace health strategies, including shift-schedule optimization, metabolic health monitoring, occupational stress management, and sleep health support.

## Data Availability

The raw data supporting the conclusions of this article will be made available by the authors, without undue reservation.
