# Supplementary material for: Association between night-shift frequency and metabolic syndrome among healthcare workers: a moderated mediation analysis of occupational stress and sleep quality
Source: Front Public Health. 2026 Sep 18;14:1940688. doi: 10.3389/fpubh.2026.1940688 (PMC13630782; doi:10.3389/fpubh.2026.1940688)
Supplement: Supplementary file 1 [file Table_1.docx]

Supplementary Material

# 1 Supplementary Data

# 2 Supplementary Figures and Tables

## 2.1 Supplementary Figures

**Supplementary Figure 1.** **Forest plot of exponentiated conditional indirect effects of night**-**shift frequency on metabolic syndrome through occupational stress across different levels of sleep quality**

Note: Conditional indirect effects were exponentiated and presented as odds ratios (ORs). Error bars represent 95% confidence intervals, and the dashed vertical line indicates OR = 1. Higher sleep quality scores indicate poorer sleep quality.

## 2.2 Supplementary Tables

**Supplementary Table 1 Sensitivity analysis of the association between night shift frequency and metabolic syndrome**

| Analysis method | Night‑shift frequency grouping | *OR* (95% *CI*) | *P* |
| --- | --- | --- | --- |
| Trend analysis | Ordinal variable | 3.344 (2.102~5.542） | <0.001 |

Note: Night-shift frequency was coded as an ordinal variable by assigning values of 0, 1, and 2 to 0, 1–3, and ≥4 night shifts/month, respectively, and was then included in the model. Models were adjusted for the same covariates as in the main analysis model.

**Supplementary Table 2 Interaction between sleep quality and night-shift frequency in relation to occupational stress**

| Interaction term | *β* | Standard error | *t* | *P-value* | 95% *CI* |
| --- | --- | --- | --- | --- | --- |
| 1-3 night shifts/month × Sleep quality score | 0.017 | 0.008 | 1.977 | 0.048 | 0.000-0.033 |
| ≥8night shifts/month × Sleep quality score | 0.038 | 0.007 | 5.239 | <0.001 | 0.024-0.052 |

Table S3 Poisson regression with robust variance analysis of the associations of night-shift frequency and occupational stress with MetS [PR (95% CI)]

Poisson regression with robust variance was further used to evaluate the association between night-shift frequency and MetS. Compared with participants reporting 0 night shifts per month, no statistically significant association was observed for those reporting 1-3 night shifts per month across all models, whereas the association between ≥4 night shifts per month and MetS remained statistically significant across the different adjustment models. In Model 4, the PR for MetS was 3.663 (95% CI: 2.117-6.338) for the ≥4 night shifts per month group and 0.698 (95% CI: 0.290-1.682) for the 1-3 night shifts per month group. The direction of the associations estimated using Poisson regression with robust variance was consistent with that obtained from logistic regression, although the effect estimates were smaller in magnitude. Detailed results are presented in Supplementary Table 3.

| Variables | Model 1 | Model 2 | Model 3 | Model 4 |
| --- | --- | --- | --- | --- |
| Sex | - | 0.510 (0.371-0.703)^***^ | 0.563 (0.373-0.851)^**^ | 0.536 (0.360-0.797)^**^ |
| Age (years) | - | 1.058 (1.042-1.075)^***^ | 1.096 (1.061-1.132)^***^ | 1.088 (1.054-1.123)^***^ |
| night shift frequency |  |  |  |  |
| 0 night shift/month | 1.000 | 1.000 | 1.000 | 1.000 |
| 1-3 night shifts/month | 0.544 (0.213-1.394) | 0.746 (0.301-1.845) | 0.725 (0.307-1.711) | 0.698 (0.290-1.682) |
| ≥4 night shifts/month | 3.754 (2.022-6.971)^***^ | 5.259 (2.994-9.238)^***^ | 5.549 (3.263-9.438)^***^ | 3.663 (2.117-6.338)^***^ |
| Occupational stress (ERI ratio) | - | - | - | 1.161 (1.102-1.222)^***^ |

Note: Poisson regression with robust variance was used to estimate prevalence ratios (PRs). Model 1 was unadjusted. Model 2 was adjusted for age and sex. Model 3 was further adjusted for educational level, ethnicity, physical activity, occupational category, healthy diet score, professional title, marital status, smoking, alcohol consumption, annual income, and family history of metabolic diseases. Model 4 was additionally adjusted for the ERI ratio. Occupational stress was reported as the PR corresponding to each 0.1-unit increase in the ERI ratio. *P < 0.05, **P < 0.01, ***P < 0.001.
